# Chloroplast Genome of *Lithocarpus* *dealbatus* (Hook.f. & Thomson ex Miq.) Rehder Establishes Monophyletic Origin of the Species and Reveals Mutational Hotspots with Taxon Delimitation Potential

**DOI:** 10.3390/life12060828

**Published:** 2022-06-02

**Authors:** Rahul Gunvantrao Shelke, Rudra Prasad Banerjee, Babita Joshi, Prem Prakash Singh, Gopal Ji Tiwari, Dibyendu Adhikari, Satya Narayan Jena, Saroj Kanta Barik

**Affiliations:** 1 CSIR-National Botanical Research Institute, Rana Pratap Marg, Lucknow 226001, India; rahul.sg98@gmail.com (R.G.S.); rudrabanerjee1042@gmail.com (R.P.B.); babita310591@gmail.com (B.J.); prem12flyhigh@gmail.com (P.P.S.); gopalnbri@gmail.com (G.J.T.); adhikari.dibyendu@nbri.res.in (D.A.); 2 Academy of Scientific and Innovative Research (AcSIR), Ghaziabad 201002, India

**Keywords:** chloroplast genome, repeat elements, RNA editing, phylogenomics, mutational hotspots

## Abstract

There is phylogenetic ambiguity in the genus *Lithocarpus* and subfamily Quercoideae (Family: Fagaceae). *Lithocarpus dealbatus*, an ecologically important tree, is the dominant species among the Quercoideae in India. Although several studies have been conducted on the species’ regeneration and ecological and economic significance, limited information is available on its phylo-genomics. To resolve the phylogeny in Quercoideae, we sequenced and assembled the 161,476 bp chloroplast genome of *L. dealbatus*, which has a large single-copy section of 90,732 bp and a small single-copy region of 18,987 bp, separated by a pair of inverted repeat regions of 25,879 bp. The chloroplast genome contained 133 genes, of which 86 were protein-coding genes, 39 were transfer RNAs, and eight were ribosomal RNAs. Analysis of repeat elements and RNA editing sites revealed interspecific similarities within the *Lithocarpus* genus. DNA diversity analysis identified five highly diverged coding and noncoding hotspot regions in the four genera, which can be used as polymorphic markers for species/taxon delimitation across the four genera of Quercoideae viz., *Lithocarpus*, *Quercus*, *Castanea*, and *Castanopsis*. The chloroplast-based phylogenetic analysis among the Quercoideae established a monophyletic origin of *Lithocarpus*, and a closer evolutionary lineage with a few *Quercus* species. Besides providing insights into the chloroplast genome architecture of *L. dealbatus*, the study identified five mutational hotspots having high taxon-delimitation potential across four genera of Quercoideae.

## 1. Introduction

*Lithocarpus dealbatus* (Hook.f. & Thomson ex Miq.) Rehder, commonly known as stone oak, is a wild tree species and belongs to the subfamily Quercoideae under the family Fagaceae. Quercoideae consists of seven genera, viz. *Castanea*, *Castanopsis*, *Chrysolepis*, *Lithocarpus*, *Notholithocarpus*, *Quercus*, and *Trigonobalanus*, with 1088–1135 species (The plant list, http://www.theplantlist.org/, accessed on 8 May 2021). The other subfamily, Fagoideae, is monogeneric with the genus *Fagus* and has 11–14 species. *Lithocarpus* is the second-largest genus of the family Fagaceae, with ~330 species, most of which are distributed in the moist/wet evergreen forests of Southeast Asia (The plant list, http://www.theplantlist.org/, accessed on 6 May 2021). In India, the genus *Lithocarpus* is represented by 17 species [1,2], of which *L. dealbatus* is the dominant species distributed in the elevation range of 1000–1500 m above sea level. The species is found in the montane subtropical and temperate forests of the Eastern Himalayas and in the hills of northeastern India, and attains a height up to 30 m [3,4,5]. The species performs several ecological functions with high standing biomass and a variety of mutualistic interactions with ectomycorrhizal fungi, gall-forming insects, and seed-dispersing vertebrates [6]. The species is mainly used as fuelwood and its acorns are eaten by rodents. The hoarding of acorns by scatter-hoarding Sciuridae (squirrels) helps the species in wide seed dispersal, maintenance of the soil-seed bank, and regeneration of the species in the forest [7,8,9,10,11,12].

Interspecific hybridisation within the family often poses challenge for species delimitation, and exhibits conflicting phylogeny [13,14,15]. The universal standard plastid markers such as *mat*K, *rbc*L, *trn*H*-psb*A, etc. do not provide a sufficient number of variable sites and phylogenetic signals to resolve the phylogeny in Quercoidaeae [16,17,18]. The use of a few nuclear markers such as ITS1 and ITS2 or their combinations for inferring Fagaceae phylogeny by earlier workers did not yield a resolved discrimination [16,17]. On the other hand, the chloroplast genome has been successfully used for establishing phylogenetic relationships, as well as to resolve taxonomic discrepancies in *Castanea* and *Castanopsis* [16]. Pang et al. (2019) achieved species-level discrimination and phylogenetic resolution in *Quercus* by using chloroplast-genome sequences [17]. The chloroplast genomes have been proven effective to resolve plant phylogeny because of their highly conserved structure and recombination-free uniparental inheritance [16]. However, Yang et al. (2021) reported that due to extensive introgression and chloroplast capture in *Quercus*, chloroplast genome analysis yielded a non-monophyletic origin while nuclear genome sequencing resulted in monophyletic origin [19]. Given such conflicting reports relating to the effectiveness of plastome in establishing phylogeny of *Quercus*, it is essential to undertake empirical studies in other genera to establish its efficacy in phylogenomics. This can be best assessed in one of its close sister genera *Lithocarpus.* In addition, there is a need to characterise the sources, extent, and consequences of the conflicting phylogenomic signals in the plastome for a mechanistic understanding.

The chloroplast-genome sequences have also been used to identify the mutational hotspot regions for designing the species-specific DNA markers/barcodes. Unlike universal markers/barcodes, the mutational hotspots can better differentiate plant taxa up to the species level [15,16]. Pang et al. [17] demonstrated that the newly discovered markers based on comparative genomics were more variable than the standard plant DNA markers (e.g., *rbcL*, *trnH-psbA* and *matK*) discriminating *Quercus* species. An extremely low-resolution power of DNA barcode was reported in the 12 Italian oak species [20]. Therefore, several workers have highlighted the need to develop novel barcodes in Quercoideae species [17,21]. Researchers have demonstrated that chloroplast-genome mutations are clustered into hotspots, and these hotspot regions can be potential candidates for new DNA barcodes [22]. Such mutational hotspots in the chloroplast genome have been used in delimiting the species-specific barcodes in oak and *Castanea* species that have been designed by previous workers [17,21]. Although some specific barcodes with high discriminatory power have been reported in *Quercus*, additional new markers are required for other Quercoideae species considering their complex evolutionary background. Therefore, genomic information is essential to understand the evolutionary relationship of *Lithocarpus* genus and its species with other genera of Fagaceae.

Plant chloroplast genomes have a circular quadripartite structure with size ranging from 107 to 218 kb [21]. The chloroplast genome is uniparentally inherited and highly conserved in terms of structural organisation, gene content, and layout, thus accumulating a slow rate of evolutionary changes [23,24,25,26,27]. Like any other chloroplast genome, small single-copy (SSC) and large single-copy (LSC) sections are separated by two inverted repeats (IR) regions in *L. dealbatus* [25]. The chloroplast genome has usually 130 genes, including 80 protein-coding genes, 4 rRNA genes, and 35 tRNA genes [19]. Despite the overall structural conservation and, contraction and expansion of the IR boundaries, the chloroplast genome can lead to variations in gene content resulting in genome size variations [28]. Moreover, several mutational events take place in the genome due to insertion or deletion, single-nucleotide polymorphisms (SNP), simple sequence repeats (SSRs) and tandem repeats [28,29]. Such genome-scale variations further allow using these regions as molecular markers in diversity research, population genetics, and phylogenetic investigations [30,31,32]. The entire chloroplast genome has recently been employed instead of single-locus DNA barcode to obtain reliable evolutionary evidences [31,33].

In recent times, the advances in high-throughput sequencing technologies have resulted in an increase in chloroplast genomes in the public domain. However, genomic information on *Lithocarpus*, particularly the complete chloroplast genome in the NCBI database (https://www.ncbi.nlm.nih.gov/nuccore/?term=Lithocarpus+chloroplast%2C+complete+genome, accessed on 4 May 2021), is represented by only two species, viz. *Lithocarpus balansae* and *Lithocarpus hancei* [34]. In view of the above, we sequenced and assembled the whole chloroplast genome of *L. dealbatus* with an objective to study its architecture, determine its effectiveness in establishing species phylogeny, and assess its structural efficacy in taxon delimitation. The assembled chloroplast genome was successfully annotated and compared with the other members of Quercoideae to understand its structural variations and rearrangements. In addition, a phylogenetic tree was constructed to deduce the evolutionary relatedness of *L. dealbatus* with other members of Quercoideae. The highly divergent regions and the SSRs identified in the chloroplast genome would also help in understanding the ecological significance of the species in terms of spatial distribution and adaptability besides the evolutionary relationship of *L. dealbtus* within Fagaceae.

## 2. Materials and Methods 

### 2.1. DNA Isolation and Sequencing on MGI Platform

Fresh leaf samples of *L. dealbatus* were collected from Shillong, Meghalaya and stored immediately in liquid nitrogen. DNA extraction was performed using DNeasy Plant Pro and Plant Kits (Qiagen) from the stored leaf samples. The quality of the extracted genomic DNA was determined in 0.8% agarose gel and further quantified through NanoDrop™ One/OneC Micro volume UV-Vis Spectrophotometer (Thermofischer, Waltham, MA, USA). The genomic library was constructed following the MGI’s DNA nanoball (DNB) protocol. DNBSEQ^TM^—G400 Genetic Sequencer (MGI Tech. Co. Ltd., Shenzhen, China) platform at Imperial Life Sciences Pvt. Ltd. was used to generate PE150long reads.

### 2.2. Chloroplast-Genome Assembly

The quality of MGI reads was checked through the FastQC program (https://www.bioinformatics.babraham.ac.uk/projects/fastqc, accessed on 8 November 2019). The clean reads were further used to de novo assemble the whole chloroplast genome via Novoplasty assembler by keeping the default parameter, except Kmer length 39 and *rbcL* gene as a seed sequence from *L. balansae* genome (Accession # KP299291.1) [35]. Finally, the assembled chloroplast genome was confirmed by performing BLASTN against the non-redundant nucleotide database at NCBI (https://blast.ncbi.nlm.nih.gov/Blast.cgi?PAGE_TYPE=BlastSearch, accessed on 18 March 2021). 

### 2.3. Annotation and Map Drawing of Chloroplast Genome

The chloroplast genome was further annotated using the Chlorobox Geseq program, keeping *L. balansae* and inbuilt the Geseq MPI-MP genome set as a reference genome (https://chlorobox.mpimp-golm.mpg.de/geseq.html, accessed on 28 April 2021). Annotations of tRNA genes were conducted through the tRNAscan-SE tool. Manual adjustments of start and stop codon and exon-intron junctions were made in the NCBI ORF finder (https://www.ncbi.nlm.nih.gov/orffinder/, accessed on 22 April, 2021). Finally, annotations and structural features of the chloroplast genome were visualised through the OGDRAW program [36].

### 2.4. Comparative Analysis of L. dealbatus with Fagaceae Chloroplast Genome for Structural Rearrangement, Similarity, Expansion and Contraction of IR and Tandem Repeat

Comparative analysis of the *L. dealbatus* chloroplast genome was performed using the previously published genomes such as *L. balansae* (Drake) A. Camus (Accession # KP299291.1), *L. hancei* (Benth.) Rehder (Accession # MW375417.1), *Castanea henryi* (Skan) Rehder & E.H.Wilson (Accession # MH998384.1), *Castanopsis sclerophylla* (Lindl. & Paxton) Schottky (Accession # NC_044680.1), *Quercus pannosa* Hand.-Mazz. (Accession # NC_050963.1) and *Trigonobalanus doichangensis* (Accession # KF990556.1). Structural rearrangement among the chloroplast genomes was recognised through MAUVE alignment (http://darlinglab.org/mauve/mauve.html, accessed on 4 May 2021). The mVISTA program was employed to determine the similarity between the compared chloroplast genomes using the Shuffle-LAGAN model by keeping *L. dealbatus* as a reference (http://genome.lbl.gov/vista/mvista/submit.shtml, accessed on 10 May 2021). The expansion and contraction of IR junctions in chloroplast genomes were analysed and displayed through the IRscope tool (https://irscope.shinyapps.io/irapp, accessed on 12 May 2021). Tandem repeats were identified through the REPuter program using the minimum repeat size 30 and hamming distance 3 (https://bibiserv.cebitec.uni-bielefeld.de/reputer, accessed on 15 May 2021). MISA tool was employed to recognise SSRs with the minimum repeats 10 for mononucleotide, 5 for dinucleotide, 4 for trinucleotide, and 3 for tetra-, penta-, hexa-, septa-, octa-, nona-, and decanucleotide, respectively (http://pgrc.ipk-gatersleben.de/misa/misa.html, accessed on 18 May 2021). We employed the Predictive RNA Editor for Plants (PREP) program to recognise the RNA editing in 35 reference genes (http://prep.unl.edu/, accessed on 24 May 2021). 

### 2.5. DNA Diversity and Ka/Ks Analysis in Lithocarpus

Divergence across the coding and noncoding regions in the *Lithocarpus*, *Quercus*, *Castanea*, and *Castanopsis* genus was determined using DnaSP v5.0 (Universitat de Barcelona, Barcelona, Spain) (http://www.ub.edu/dnasp/, accessed on 8 June 2021). MEGA X tool was used to determine the Ka/Ks ratio of protein-coding regions (https://www.megasoftware.net/, accessed on 18 June 2021). 

### 2.6. Phylogenetic Analysis and Estimation of the Divergence Time

Phylogenetic study was performed using the chloroplast genomes of Quercoideae genomes available in the public database. We selected two chloroplast genomes from the genus *Fagus*, namely *F. crenata* and *F. japonica*, as outgroups. Since structural rearrangements, gene content and direct alignment of chloroplast genomes are challenging, we employed the HomBlocks tool to determine locally collinear blocks (LCBs) present in chloroplast genomes for alignment [37]. Unaligned sequences were trimmed using the Gblocks program embedded in the HomBlocks pipeline. The Model test calculated the best substitution model in the MEGA tool suggested by the Akaike information criterion (AIC). Finally, the GTR+G+I model was chosen to construct the phylogenetic tree using the maximum-likelihood (ML) method in the MEGA X tool (https://www.megasoftware.net/, accessed on 8 August 2021). The branch support values for each were calculated based on 500 bootstraps. The phylogenetic tree was coloured and represented using the iTOL web server (https://itol.embl.de/, accessed on 13 August 2021). 

A timetree was inferred using the ReltimeML-option in MEGA X. The same species used in our previous analysis such as *F. crenata* and *F. japonica* were constrained to be the outgroup in this divergence-tree analysis. The reference-node age was obtained by the divergence time of *Quercus ciliaris–T. doichangensis* (11.1–57.9 million years ago) and *Q. ciliaris–C.henryi* (6.0–49.0 million years ago) (http://www.timetree.org/, accessed on 10 November 2021).

## 3. Results

### 3.1. Assembly of Chloroplast Genome and Annotation 

#### 3.1.1. *L. dealbatus* Chloroplast-Genome Assembly and Architecture

**Figure 1:**
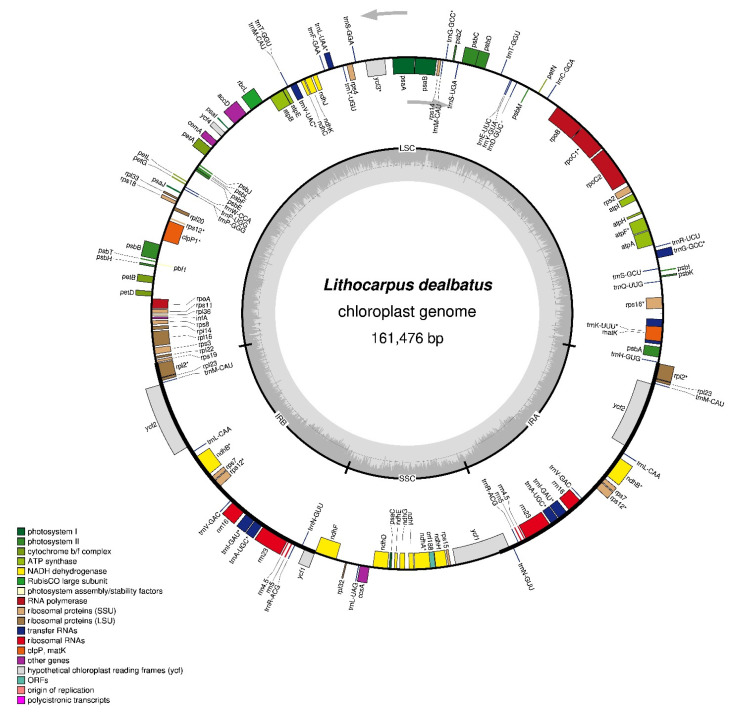
Circular map of *Lithocarpus dealbatus* chloroplast genome representing the annotated genes in different colours according to their specific functions. The genes present on the inner circular map were transcribed clockwise, and those situated outside were transcribed anticlockwise. The darker grey colour inside the circle shows the GC content, while the lighter grey colour indicates the AT content. The chloroplast-genome borders were demarcated as LSC, SSC, IRA, and IRB regions. * represents the intron in genes.

About 47 Gb data with more than 156,718,852 adapter clean short PE reads were used for de novo chloroplast-genome assembly. The total length of the assembled *L. dealbatus* chloroplast genome was 161,476 bp with an average coverage of 1494× (Figure 1). The complete chloroplast genome exhibits a typical quadripartite structure, comprising a pair of IR (IRA and IRB) regions (25,879 bp) divided by an SSC region (18,987 bp) and an LSC region (90,732 bp). The overall GC content of the genome was 36.7%, while the GC content of LSC, SSC, and IR regions were 34.6%, 30.9%, and 42.7%, respectively.

Homology searched through the BLASTN program revealed a high sequence similarity of the *L. dealbatus* chloroplast genome with the *L. hancei* and *L. balansae* chloroplast genome. A high-quality chloroplast-genome sequence was finally submitted to the Genbank, NCBI database under the accession number MZ322408. 

#### 3.1.2. Chloroplast-Genome-Encoding Genes 

The whole chloroplast genome of *L. dealbatus* encodes 133 genes, consisting of 86 protein-coding genes, 39 transfer RNA(tRNA), and 8 ribosomal RNA (rRNA) genes (Table 1). Among 133 genes, 8 protein-coding genes (*ndhB*, *psbD*, *rpl2, rpl23*, *rps7*, *rps12*, *ycf1* and *ycf2*), 9 tRNA, and 4 RNA genes were duplicated in the genome (Table 2). Altogether, 13 protein-coding and 8 tRNA genes contained an intron, in which two genes (*ycf3* and *clpP*) harboured a double intron. In addition, *rps12* was identified as a trans-spliced gene in the genome. However, *trnk-UUU* has an intron encompassing the *matK* gene. Each of the IR regions harboured seven protein-coding genes, seven tRNA and four rRNAs.

#### 3.1.3. Substitution Rate of Protein-Coding Genes of *Lithocarpus*

**Figure 2:**
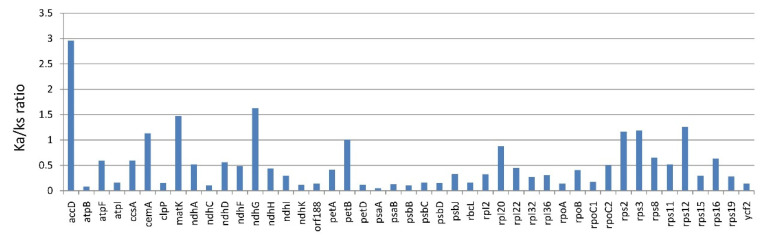
The comparative analysis of Ka/Ks ratio from *Lithocarpus* chloroplast genomes for individual genes.

The nonsynonymous substitution (Ka) to synonymous substitution (Ks) ratios were calculated to understand the evolutionary pressure on the protein-coding sequences. A total of 79 shared protein-coding genes across all three *Lithocarpus* chloroplast genomes were utilised to calculate Ka/Ks ratios (Figure 2). The current analysis shows that the Ka/Ks ratio for 34 genes was zero. However, 37 genes revealed a Ka/Ks ratio between the range of 0 and 1.0, which shows that these genes were under the purifying selection. The Ka/Ks ratios of eight genes (*accD*, *cemA*, *matK*, *ndhG*, *petB*, *rps2*, *rps3* and *rps12*) were greater than 1, indicating the positive selection acting on protein-coding genes. The average Ka/Ks ratio for 45 genes was 0.52.

### 3.2. Comparative Chloroplast Genomes in Quercoideae 

#### 3.2.1. Comparison of Quercoideae Chloroplast Genomes

**Figure 3:**
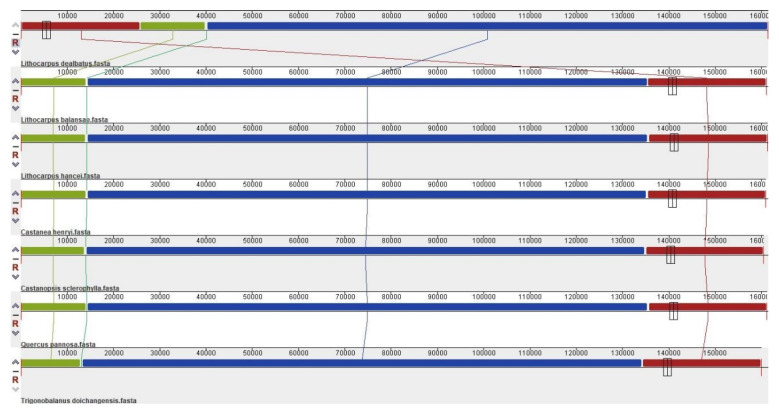
MAUVE alignment of *L. dealbatus* chloroplast genome against some of the representative species from Quercoideae. Within each of the alignments, the local colinear blocks indicate the identical conserved regions represented by the same colour and are joined by lines.

**Figure 4:**
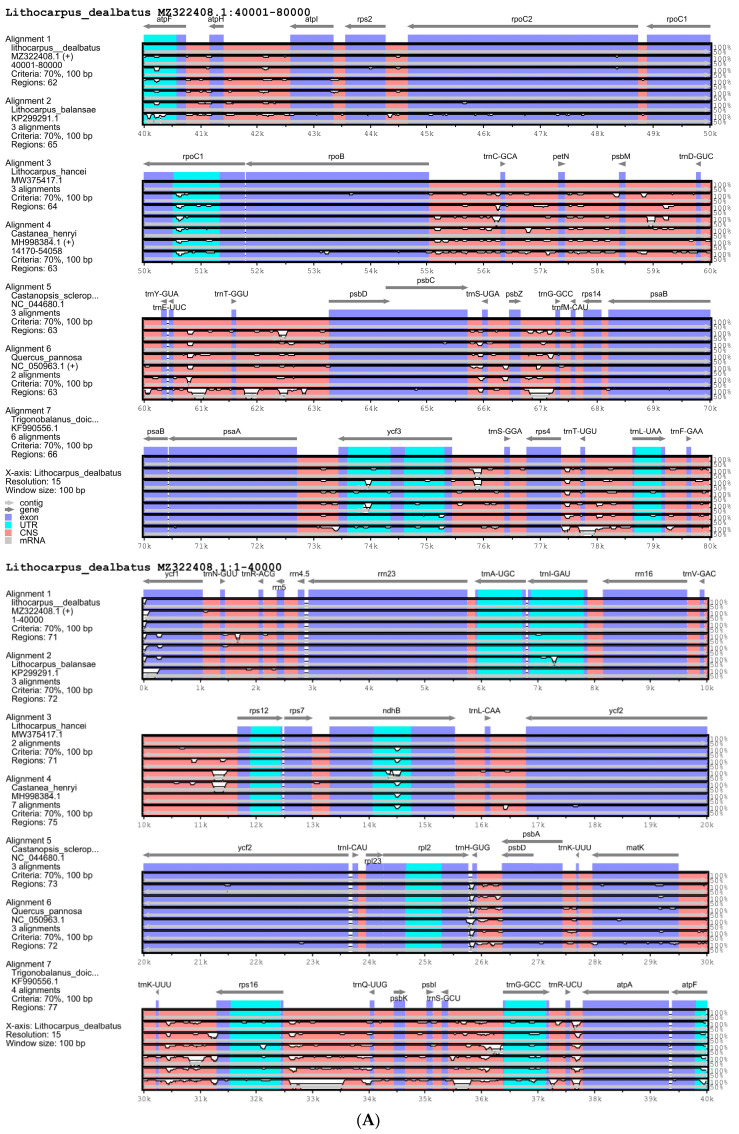

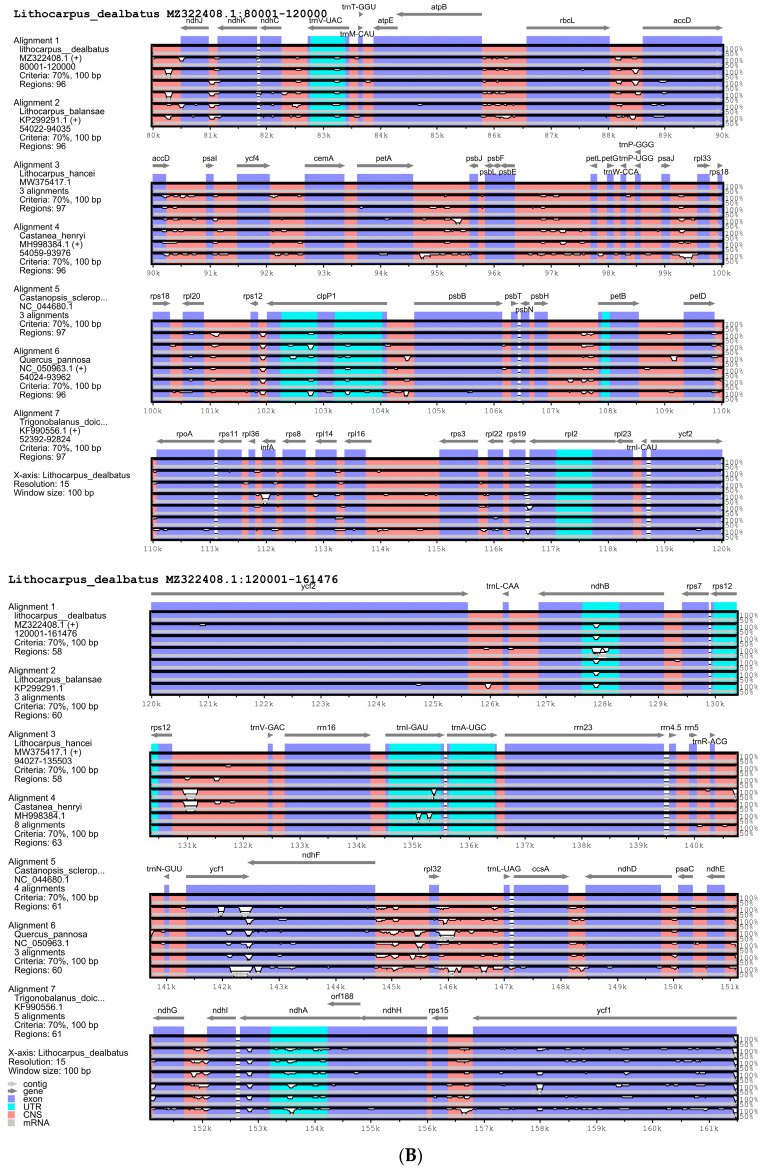
Sequence alignment of Quercoideae chloroplast genomes by mVISTA, with annotated *L. dealbatus* chloroplast genome as the reference, (**A**) Sequence alignment of 1 to 80,000 bp, (**B**) Sequence alignment of 80,001 to 161,476 bp.

Four locally collinear blocks were identified in *L. dealbatus* and the six other previously published Quercoideae members using the multiple-genome-alignment tool MAUVE (Figure 3). Overall, the synteny of the gene order was similar in all genomes, except for an inversion of about 275 bp in the LSC region of *T. doichangensis* (Figure S1). The inversion explicitly occurred in the intronic region of the *atpF* gene. The mVista genome alignment showed that the genic regions were mostly conserved, with a few exceptions (Figure 4). Overall, the *Lithocarpus* genome showed a higher level of nucleotide identity than that of *Castanea*, *Castanopsis*, and *Quercus*. In the present investigation, the *ycf1* gene revealed significant variation among the seven compared genomes. Intronic regions revealed a greater level of divergence comparison to the un-translated region (UTR) and genic regions.

**Figure 5:**
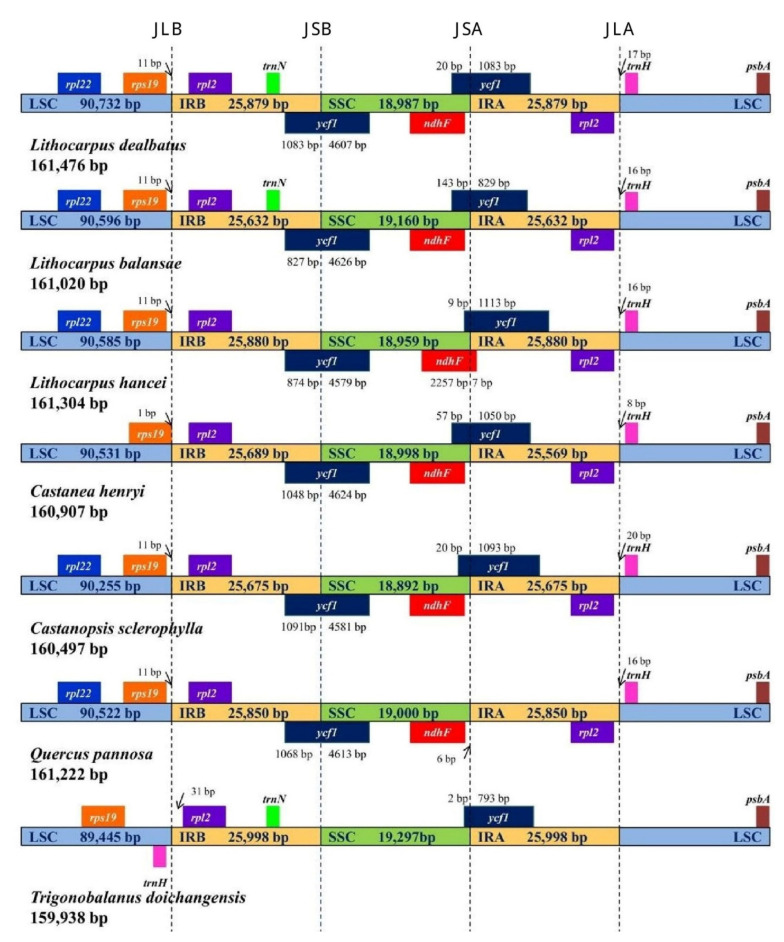
Details of junction positions between inverted repeat regions and small and large single-copy regions across the compared Quercoideae genomes. Each gene is depicted in different colours. The genes present on top are transcribed on the positive strand, whereas those present below are transcribed on the negative strand. The complete genome size of each species is mentioned on the left side.

The variation in size of chloroplast genomes is often due to the expansion and contraction of IR junctions in higher plants. Hence, we analysed and compared the location of the IR border and their adjacent genes among the seven studied chloroplast genomes (Figure 5). In *L. dealbatus*, the *ycf1* gene was detected at IRB/SSC junction with 4607 bp inside SSC and 1083 bp inside the IRB region. On the other hand, the other partially duplicated copy of the *ycf1* gene was found at IRA/SSC junction with 1083 bp inside IRA and 20 bp inside the SSC region. A similar trend was observed in all the compared genomes except in *Q. pannosa* and *T. doichangensis*, where *ycf1* was missing at IRA/SSC or IRB/SSC junctions. The size of the *ycf1* gene in *L. dealbatus* was in the range of 1103 bp to 5690 bp, while it was 795 bp in *T. doichangensis* and 5681 bp in *Q. pannosa* genome. The *ndh*F gene was positioned near the IRA/SSC region in the compared genomes, except in *T. doichangensis*. The *ycf1* gene overlapped with *ndhF* gene at IRA/SSC junctions in three genomes, viz., *L. hancei*, *Castanopsis sclerophylla*, and *Castanea henryi*. Two genes, namely *rps*19 and *rpl*2, were spotted completely inside the LSC and IRB regions on either side of the IRB/LSC junction. Another duplicated copy of the *rpl2* gene was situated entirely inside the IRA region and was absent in *T. doichangensis*. We observed the position of the *trnH* gene within LSC region in all of the seven compared genomes.

#### 3.2.2. Repeat Sequences and Its Comparative Analysis

**Figure 6:**
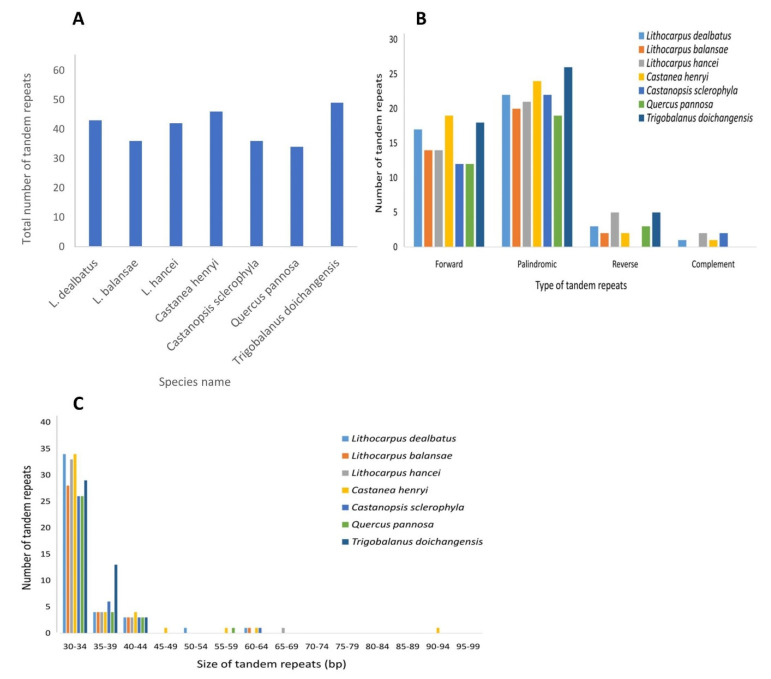
Comparative analysis of tandem repeats across compared genomes, (**A**) Total number of tandem repeats distributed across the chloroplast genomes of seven studied species (**B**) Distribution of tandem repeats based upon their type in the chloroplast genome, (**C**) Frequency of tandem repeats based on their size in the chloroplast genome.

The chloroplast genome of *L. dealbatus* comprised 43 repeat elements, of which 17 were forward, 22 were palindromic, 3 were reverse, and 1 was a complement repeat (Figure 6A,B). Overall, the total number of tandem repeats varied between 34 in *Q. pannosa* and 49 in *T. doichangensis* (Figure 6A). Comparative analysis of tandem repeats in seven studied genomes revealed that most repeats belonged to palindromic type, having maximum repeats in *T. doichangensis* (26) and minimum in *Q. pannosa* (19) (Figure 6B). Followed by palindromic type, the forward repeats had the highest number in *C. henryi* (19) and lowest in *C. sclerophylla* and *Q. pannosa*, each with 12 repeats. The reverse repeats were absent in *C. sclerophylla*, while these were highest in *L. hancei* and *T. doichangensis* (5 each). Similarly, a small number of complement repeats were observed in *L. hancei* (2), *C. henryi* (1) and *C. sclerophylla* (2), and it was absent in *L. balansae*, *Q. pannosa* and *T. doichangensis.* The maximum number of repeats had 30–34 bp, while only a few had 45–64 bp (Figure 6C).

**Figure 7:**
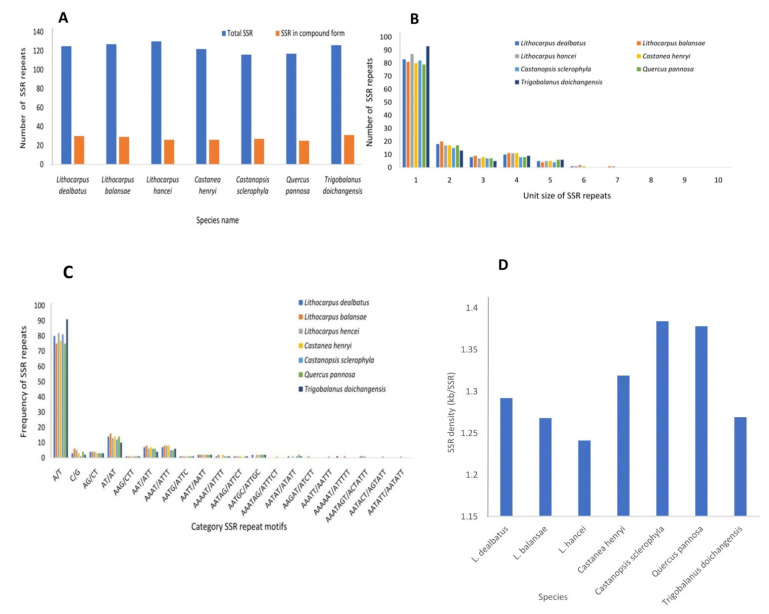
Category of SSRs in the compared Quercoideae genomes. (**A**) Distribution of SSRs in simple and compound form; (**B**) Representation of SSRs based on their unit size; (**C**) Distribution of SSRs based upon the repeat motifs; (**D**) Density of SSR repeats showed in the chloroplast genomes of seven studied species.

We mined 125 simple and 30 compound SSRs in the chloroplast genome of *L. dealbatus* (Figure 7A). Among 125 simple SSRs, 83 were mononucleotides (66.4%), 18 were di-nucleotides (14.4%), 8 were tri-nucleotides (6.4%), 10 were tetra-nucleotides (8%), 5 were penta-nucleotides (4%), and only 1 was a hexa-nucleotide (0.8%) (Figure 7B). Comparative analysis of SSRs among the studied species revealed that *L. balansae*, *L. hancei*, *C. henryi*, *C. sclerophylla*, *Q. pannosa*, and *T. doichangensis* contained a total of 127, 130, 122, 116, 117, and 126 SSRs, respectively.

The highest percentage of mononucleotide SSRs was in *T. doichangensis* (73.8%), followed by *C. sclerophylla* (70.6%), and the least was in *L. balansae* (63.7%). Similarly, the percentage of dinucleotide SSRs was maximum in *L. balansae* (15.7%), and minimum in *T. doichangensis* (10.3%). The most abundant mononucleotide SSRs had A/T repeat motifs and C/G repeat motifs were rare. The highest number of A/T rich repeats was in *T. doichangensis* (91), followed by *L. hancei* (82), and the least was recorded in *L. balansae* and *Q. pannosa* with 75 repeats each. Similarly, the dinucleotide repeats were rich in AT/AT compared to AG/CT and were highest in *L. balansae* (16) and lowest in *T. doichangensis* (10) (Figure 7C). SSR density was relatively greater in *C. sclerophylla* and *Q. pannosa* (~1.4 kb/SSR) than that of the other species. The least SSR density was recorded in *L. hancei* (~1.23 kb/SSR) (Figure 7D).

#### 3.2.3. Nucleotide Diversity and Mutational Hotspots

**Figure 8:**
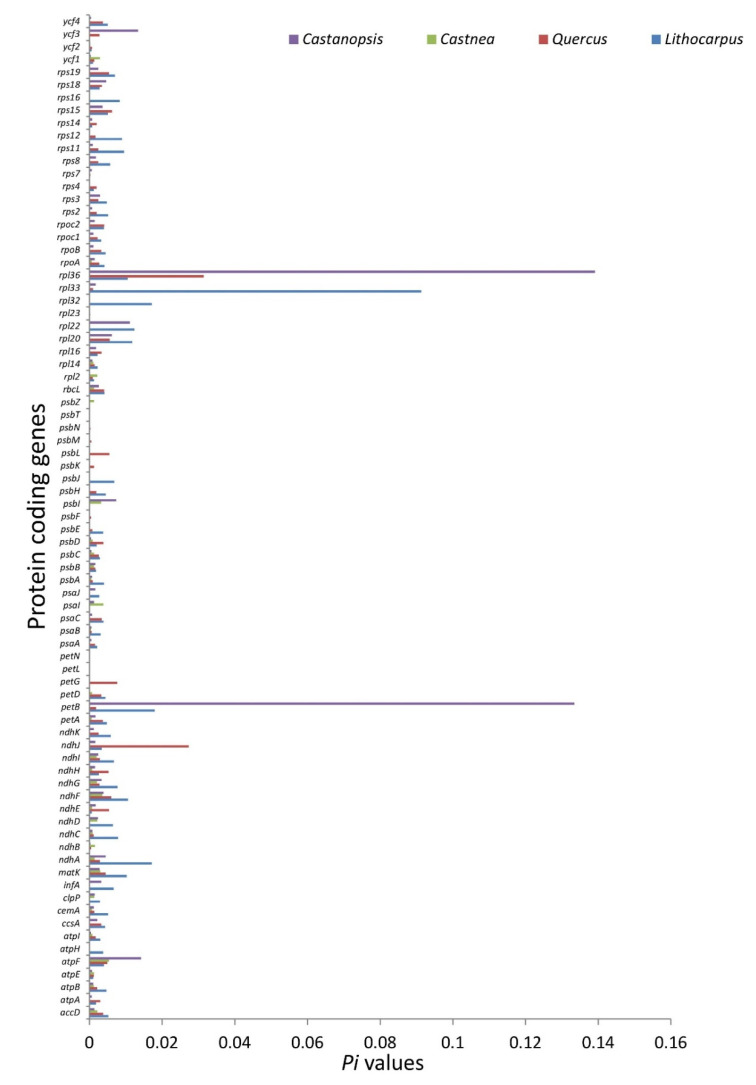
Comparison of the DNA diversity (*Pi*) values of protein-coding regions in *Lithocarpus*, *Quercus*, *Castanea*, and *Castanopsis*.

Comparative analysis of the nucleotide variation in coding and noncoding regions was carried out to identify the hotspot regions in *Lithocarpus*, *Quercus*, *Castanea*, and *Castanopsis* chloroplast genomes. We found high divergence in noncoding regions than the protein-coding regions. The nucleotide diversity (*Pi*) for protein-coding regions ranged from 0.00059 (*ycf2*) to 0.09132 (*rpl33*) for *Lithocarpus* (Figure 8), while for *Quercus* it ranged from 0.0002 (*rpl23*) to 0.03146 (*rpl36*), in *Castanea* it ranged from 0.000256 to 0.00533, and in *Castanopsis* it ranged from 0.000175 to 0.13913 (Figure 8). *Castanopsis* had the highest average nucleotide diversity (0.006989) for protein-coding genes, followed by *Lithocarpus* (0.0066), *Quercus* (0.0034), and *Castanea* (0.001617). We observed five highly diverged coding regions in each member of Quercoideae, such as *rpl33*, *petB*, *rpl32*, *ndhA*, and *rpl22* in *Lithocarpus*; *rp136*, *ndhJ*, *petG*, *rps15*, and *ndhF* in *Quercus*; *atpF, psaI, ndhF, psbI*, and *matK* in *Castanea*; and *rpl36*, *petB*, *atpF*, *ycf3*, and *rpl22* in *Castanopsis*. However, across the four studied members of Quercoideae, the coding loci such as *rpl36*, *rpl33*, *ndhJ*, *atpF*, and *ndhA* were highly diverged.

The nucleotide diversity (*Pi*) on the other hand for noncoding regions ranged between 0.000278 (*ycf2_trnL-CAA*) and 0.04390 (*trnH-GUG_psbA*) for *Lithocarpus* (Figure S2A), 0.000536 (*ndhB_rps7*) and 0.013736 (*petA_psbJ*) for *Quercus* (Figure S2B), 0.000605 (*ycf4_cemA*) and 0.028562 (*trnH_GUG_psbA*) for *Castanea* (Figure S2C), and 0.000691 (*ndhG_ndhI*) and 0.027903 (*rbcL_accD*) for *Castanopsis* (Figure S2D). Overall, *Lithocarpus* had the greatest average nucleotide diversity (0.009315) of noncoding regions, followed by *Castanopsis* (0.006118), *Quercus* (0.005901), and *Castanea* (0.005159). The highly diverged noncoding regions such as *trnH-GUG_psbA*, *rbcl_accD*, *cssA_ndhD*, *trnF-GAA_psbA*, and *psbK_psbI* were found in *Lithocarpus*, followed by *Quercus* (*petA_psbJ, atpF_atpH, psaC_ndhE, ycf4_cemA* and *atpI_rps2*), *Castanea* (*ndhG_ndhI*, *rbcL_accD*, *psbC_trnS_UGA*, *ccsA_ndhD* and *psaJ_rpI33*), and *Castanopsis* (*rbcL_accD*, *ccsA_ndhD*, *psbK_psbI*, *psaJ_rpl33* and *psaI_ycf4*). 

#### 3.2.4. RNA Editing Sites and Its Comparative Analysis in Quercoideae 

**Figure 9:**
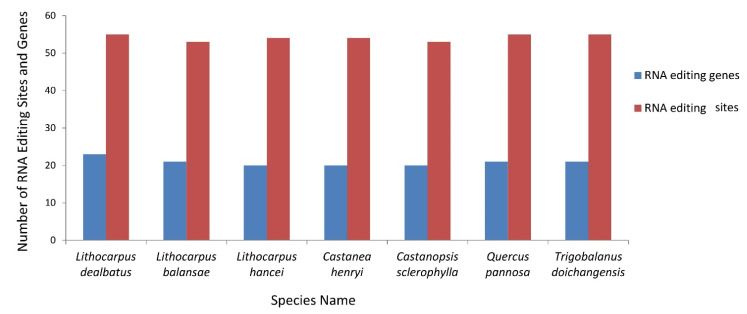
Distribution of RNA editing sites in genes across the compared genomes.

The RNA editing sites in the *L. dealbatus* chloroplast genome were predicted using the program PREP suite. Overall, the programme predicted 55 editing sites in the 23 protein-coding genes (Figure 9). All the substitution sites were from nucleotide base C to T. The analysis showed that most conversions were from the amino acid serine to leucine, followed by proline to leucine. The highest 42 substitutions occurred at the second codon position and 13 substitutions occurred at first codon positions. Of the 55 editing sites, we observed the highest editing sites in *ndhB* (10 sites), followed by *ndhD* (5 sites) and *rpoB* (5 sites) genes. Fifty-two RNA editing sites in the *L. dealbatus* chloroplast genome were responsible for changes in hydrophobic amino acids such as isoleucine, leucine, proline, phenylalanine, methionine, valine, tyrosine, cysteine, and tryptophan. 

Comparative analysis of RNA editing sites among the Quercoideae genomes revealed that *L. balansae* had 53 sites in 21 genes, *L. hancei* had 54 sites in 20 genes, *C. henryi* had 54 sites in 20 genes, *C. sclerophylla* had 53 sites in 20 genes, *Q. pannosa* had 55 sites in 21 genes, and *T. doichangensis* had 55 sites in 21 genes (Figure 9). An analysis of gene-specific RNA editing sites in different Quercoideae members revealed that the *psaI* gene in *L. dealbatus*, *ycf3* gene in *Q. pannosa*, *rpl20* gene in *L. balansae*, and *psbB* gene in *T. doichangensis* hosted the respective RNA editing sites. However, the *L. dealbatus* genome showed more RNA editing genes than the other genomes (Table S1). 

### 3.3. Chloroplast-Based Phylogenetic Analysis in Quercoideae 

#### 3.3.1. Phylogenetic Analysis of Quercoideae 

**Figure 10:**
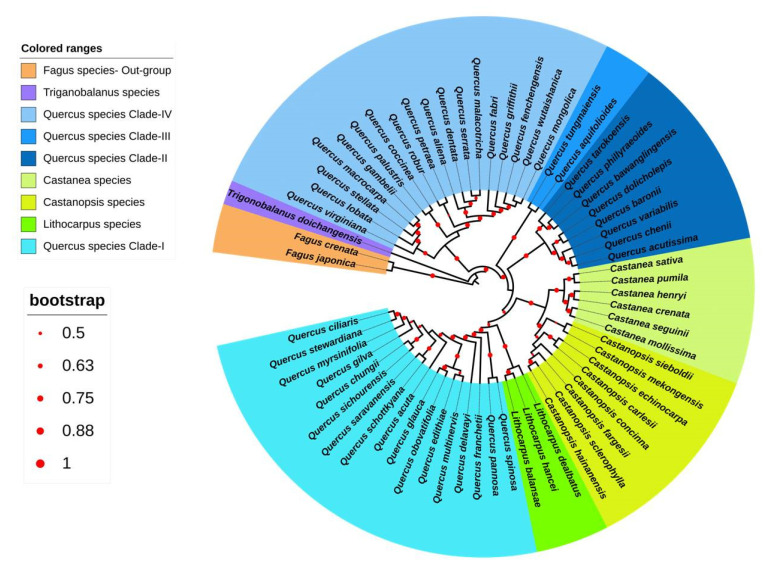
ML phylogenetic-tree construction based on the whole chloroplast genome of Quercoideae species by keeping *Fagus* species as an outgroup.

This study constructed a phylogenetic tree based on 95,904 bp nucleotide alignment by employing the GTR+G+I as the best-fit model. The topology of phylogenetic tree was constructed following ML method. The chloroplast genomes of 63 Quercoideae members were included by keeping two *Fagus* species as an outgroup (Figure 10). The phylogenetic tree showed that three *Lithocarpus* species, viz. *L. dealbatus*, *L. hancei*, and *L. balansae* formed a separate group sharing a common node with Clade-I of *Quercus* species. Among the three *Lithocarpus* species, *L. dealbatus* was closer to *L. hancei* and *L. balansae*. We observed that the *Quercus* genus was the most diverse among the Quercoideae, forming four different clades in the phylogenetic tree. On the other hand, *Castanopsis* and *Castanea* formed two separate monophyletic groups and shared a common node. *T. doichangensis* occupied the basal position in the phylogeny of Quercoideae.

#### 3.3.2. Estimated Lineage (Stem) Divergence Time for Quercoideae Members

Molecular-clock analysis suggests that the estimated lineage (stem) divergence time for Quercoideae members such as *Lithocarpus-Quercus*-*Castanopsis-Castanea* was ~37.28 Mya (Figure S3). However, *Lithocarpus* evolved ~22.80 Mya from one clade of *Quercus*, while *Castanea-Castanopsis* evolved ~23.63 Mya from *Lithocarpus-Quercus* (1st clade). The estimated lineage divergence time for *Castanea* was ~21.02 Mya. The other clades of *Quercus* (II, III, and IV) had lineage divergence times more than ~23.90 Mya (Figure S3).

## 4. Discussion 

*Lithocarpus*, the second-largest genus of the Quercoideae subfamily under Fagaceae, is dominant in many subtropical and temperate forests, contributing greatly to ecosystem structure, function, and services [38]. *L. dealbatus* is the dominant species in Indian montane subtropical and temperate forests. Although it is an ecologically and economically important species [6,7,9,10,11], no genomic information on *L. dealbatus* is available. The availability of chloroplast genomic resources is critical for unraveling the genetic architecture, evolutionary relationship, and taxon delimitation. In this context, we assembled the *L. dealbatus* chloroplast genome and carried out a comparative analysis with six other members of Quercoideae (available in public domain) to comprehend the structural architecture of the chloroplast genome and phylogenomic analysis of Quercoideae. 

The *L. dealbatus* chloroplast genome size was 161,476 bp, which was in the expected range for most angiosperm chloroplast genomes (107–218 kb) [21]. The typical quadripartite structure of the genome was consistent with other angiosperm chloroplast genomes [18]. The genome displayed a low GC content (36.7%), which is similar to other reported genomes from Fagaceae family [17,18]. Due to high GC content of rRNA genes, IR regions had greater GC content (42.7%) than both the LSC and SSC regions [17,18]. Despite minor variations, the gene content was nearly conserved in the three studied *Lithocarpus* chloroplast genomes. 

The presence or absence of protein-coding genes constituted a notable difference among the studied genomes. We observed the loss of the *psbZ* gene in *L. balansae* and *L. hancei* genomes. Similar loss of *psbZ* gene was also identified in *C. sclerophylla*, *T. doichangensis* and other land plants [39]. In contrast, no loss of protein-coding genes was noticed in the *L. dealbatus* chloroplast genome. Absence of the *infA* gene was also reported in a few angiosperm chloroplast genomes [40]. In the present investigation, the *infA* gene was absent in *C. henryi* and *T. doichangensis.* However, on several occasions, the missing chloroplast genes were reported to be integrated into the nuclear genome [21,25].

The chloroplast genome is known to be conserved in terrestrial plants. However, specific structural changes in the genome, such as inversions caused by random rearrangements, have been described by earlier works [41]. Except for a tiny 275 bp inversion in the *T. doichangensis* genome, our study revealed no significant inversions in the compared genomes. Such minor inversions are common in angiosperm chloroplast genomes [35]. The mVISTA analysis revealed that the noncoding regions were more variable than protein-coding regions, which is a trend also observed in other chloroplast genomes [16,42,43]. The IR region showed higher levels of conservation than the LSC and SSC regions, which is similar to other angiosperms [16]. The level of divergence in *Lithocarpus* genomes was calculated using nucleotide diversity (*Pi*) analysis. Consistent with the divergence pattern observed in most angiosperms, greater divergence was detected in *Lithocarpus* noncoding regions [30]. The majority of the hotspot divergence regions have been previously utilised to develop potential molecular markers [29]. Our analysis indicates that these highly divergent regions could be important in discriminating the *Lithocarpus* species.

The IR regions are considered as the most conserved region in chloroplast genomes [42]. However, several studies have observed variation in chloroplast genome size and rearrangement in many plant species, which were attributed to contraction and expansion of IR regions [42,43,44]. Our study revealed similarities and dissimilarities among the IR junctions of the studied genomes. IR areas were mostly conserved across the *Lithocarpus* genomes. Nearly similar results were obtained for *Castanopsis* and *Quercus* genomes. However, the absence of the *ycf1* gene was noticed at IRA/SSC and IRB/LSC junctions in *Q. pannosa* and *T. doichangensis*, respectively. Several researchers have documented the loss of the *ycf1* gene at IR junctions in many land plants [42,45,46]. Among the studied genomes, we observed extensive rearrangements at the IR junctions in *T. doichangensis*. These variations contribute significantly to the evolution of the chloroplast-genome structure. Long and complex repeats play a pivotal role in genome rearrangement and divergence [46]. Due to high sequence variations, tandem repeats are ideal for developing physical and genetic maps [47,48]. Our results of repeat analysis revealed variations in the total number of tandem repeats among the compared genomes, which is consistent with the earlier observations in the Plantaginaceae and Schisandraceae chloroplast genomes [42,49]. Moreover, minor variation was noticed in the distribution of tandem repeats regarding type and length, similar to the distribution pattern described in other plants [42,49]. SSRs are widely distributed in the chloroplast genome and are associated with sequence rearrangement and polymorphism [42,50].

In the current study, SSR density moderately varied in the studied genomes. The highest density of SSR was observed in *C. henryi* (1SSR/1.31 kb) followed by *Q. pannosa* (1SSR/1.37 kb) and *C. sclerophylla* (1SSR/1.38 kb). The density of SSRs detected in the present investigation was greater than those found in rice and the members of Solanaceae family [51,52]. As previously reported [51], mononucleotides were the most frequent SSR repeats, followed by dinucleotide and tri-nucleotide repeats. In mononucleotide repeats, the population of A/T repeats was significantly greater than the population of G/C repeats. Similar SSR distributions have been observed in Fagaceae species and other angiosperms [53]. These findings suggest that the repeats mined in the current study can be utilised to develop molecular markers for studying population genetics, phylogeny, and for differentiation of taxon within *L. dealbatus* and other members of Fagaceae.

RNA editing is an essential post-transcriptional mechanism observed in land plants. Identifying RNA editing sites in chloroplast genes help us to comprehend the underlying regulatory process(es) and its biological significance [43,54]. We detected 53–55 RNA editing sites in 20–23 protein-coding genes across the compared genomes. *L. dealbatus* chloroplast genome exhibited the highest RNA editing genes (in 23 genes) compared to closely related *L. balansae* (in 21 genes) and *L. hancei* (in 20 genes) genome. In a broad sense, the number of RNA editing genes and sites are variable in chloroplast genomes [28]. We found three gene-specific RNA editing sites in the present investigation. However, the loss or gain of editing sites and their frequencies is an independent event that arises through mutations in RNA editing factors or stress interference [54,55,56]. Consistent with the previous reports, the highest number of editing sites were detected in the *ndhB* (10 sites) and *ndhD* (8 sites) genes [28,54]. In addition, we noticed frequent conversions at the second base position of the codon that may change the corresponding amino acid, leading to alterations in protein primary, secondary, or tertiary structures. Consequently, such modifications can play a crucial role in protein functioning [43]. Generally, most RNA editing conversions result in hydrophobic amino-acid change, thus influencing the protein structure [28,43]. Therefore, a comprehensive investigation of RNA editing sites is an inevitable exercise that requires further attention.

The occurrence of point mutations through synonymous and non-synonymous nucleotide substitutions is crucial for gene evolution [57]. The calculation of Ka/Ks ratios has been extensively used to detect evolutionary pressure acting on protein-coding genes [50,58]. Most protein-coding genes from *Lithocarpus* showed Ka/Ks ratios less than 1, which is consistent with the previous reports [47,59]. This suggests that the majority of genes are under purifying selection. However, 34 genes showed a Ka/Ks ratio of 0; such ratios were observed when the Ks values were either very low or had no substitution present between the aligned sequence [29]. In addition, we observed positive selection in eight *Lithocarpus* chloroplast genes (*accD*, *cemA*, *matK*, *ndhG*, *petB*, *rps2*, *rps3*and *rps12*) with Ka/Ks values greater than 1. These genes have also been reported for positive selection in other species [60]. Overall, the average Ka/Ks ratio (0.52) of the *Lithocarpus* clade reported in the present investigation was greater than that observed in the *Corydalis* species (0.26) [60], suggesting a possible evolutionary change in specific genes.

DNA barcoding is a fast and accurate method for identifying species by employing a short piece of genomic DNA [61]. However, no universal barcode is available hitherto that can discriminate the taxa up to species level [15,17]. Researchers have recently employed novel strategies to overcome these issues through two approaches, namely DNA hotspot regions and super-barcoding [32,33,47,62,63]. Here, we have used the first approach to suggest the DNA divergent regions for resolving taxonomic conflicts and proposed a DNA barcode in four members of Quercoideae. We suggest five highly diverged coding regions (*rpl33*, *petB*, *rpl32*, *ndhA* and *rpl22* in *Lithocarpus*; *rp136*, *ndhJ*, *petG*, *rps15*, *ndhF* in *Quercus; atpF*, *psaI*, *ndhF*, *psbI*, *matK* in *Castanea;* and *rpl36*, *petB*, *atpF*, *ycf3*,*rpl22 in Castanopsis*) and noncoding regions (*trnH-GUG_psbA*, *rbcl_accD*, *cssA_ndhD*, *trnF-GAA_psbA*, *psbK_psbI* in *Lithocarpus; petA_psbJ*, *atpF_atpH*, *psaC_ndhE*, *ycf4_cemA* and *atpI_rps2* in *Quercus*; *ndhG_ndhI*, *rbcL_accD*, *psbC_trnS_UGA*, *ccsA_ndhD*, *psaJ_rpI33* in *Castanea;* and *rbcL_accD*, *ccsA_ndhD*, *psbK_psbI*, *psaJ_rpl33*, *psaI_ycf4* in *Castanopsis)* from each genus by comparing chloroplast genome sequences. However, a comparative study among the four genera revealed five highly diverged coding regions (*rpl36*, *rpl33*, *ndhJ*, *atpF*, and *ndhA*), which could be used to resolve intergeneric discrepancies. Similar experiments were carried out in other studies identifying highly variable regions [32,47,63]. Most of the discovered barcodes in the current study were not reported as universal markers in previous studies [15,17,60]. Therefore, with these newly discovered barcodes, the taxon delimitation efficacy should enhance manifold. Further design of intra- and inter-generic barcodes would be helpful in achieving greater phylogenetic resolution.

Recently, researchers proposed the utility of the whole chloroplast genome as a “super-barcode”, which has provided a new perspective to plant identification and species delimitation [57,58]. Previous studies in *Quercus* revealed low phylogenetic resolution using universal barcodes such as *rbcL*, *matK*, and *trnH-psbA* [15,17]. Thus, an increasing number of investigations are employing the whole chloroplast genome to overcome the low resolving power of single-locus markers for evaluating phylogenetic relationships [37,62]. In the present study, resolving the phylogeny between *Lithocarpus* and other Quercoideae species could give insights into the evolutionary relationship in the Fagaceae family. *Lithocarpus* genus formed a monophyletic group in which *L. hancei* and *L. balansae* were closely associated. The loss of the *psbZ* gene in *L. hancei* and *L. balansae* genome also confirms such a phylogenetic relationship. 

*L. dealbatus* was more closely related to *L. hancei* than *L. balansae*, which is well-supported by sequence homology analysis. The *Lithocarpus* clade shared a common node with the *Quercus* species of clade-I, consistent with the high coverage and sequence similarity between some *Quercus* and *Lithocarpus* species. At the same time, molecular dating analysis revealed the divergence time for *Lithocarpus* from *Quercus* clade at ~22.80 Mya in the early Miocene Epoch. A near-similar divergence time was estimated for separating the *Quercus* and *Lithocarpus* genus based on the five genes [63]. Furthermore, phylogeny analysis suggests that *Castanea* and *Castanopsis*, two closely diverged genus, split ~21.02 Mya, which is in conformity with the previous morphological and molecular studies [31,64]. On the other hand, *T. doichangensis* was present at the basal position and showed as an early diverged genus in the Quercoideae subfamily. This is well-supported by the previous fossil records [65]. Moreover, *Quercus* appeared as the most diverse species forming four distinct clades in the phylogenetic tree, i.e., non-monophyly [31,66]. Yang et al. (2021) also suggested that chloroplast capture through hybridisation during the early diversification of Quercoideae results in the non-monophyly of *Quercus* [19]. A few previous studies also reported that chloroplast DNA spreads more freely among the geographically co-distributed species than the nuclear DNA [67,68]. Thus, it is critical to compare plastid phylogenies with nuclear genome phylogenies for a complete understanding of evolutionary history in Quercoideae. Using multiple RAD-seq datasets, previous phylogenetic studies reconstructed *Quercus* as monophyletic with moderate to high support [69,70,71]. However, all of our plastid phylogenomic analyses suggested a nonmono-phyletic origin of *Quercus*, which is consistent with earlier plastid phylogenetic research [72]. The phylogenetic relationship of oak is complex, owing to a considerable level of hybridisation, introgression, incomplete lineage sorting, and convergent evolution [73]. Thus, phylogenetic linkages estimated from plastid data often contradict with those inferred from nuclear data [67,74,75]. However, this non-monophyly could be because of endemism and allopatric speciation among *Quercus* species that may have amplified genetic and morphological variations in species evolution [18]. Our tree topology comprising *Fagus*, *Trigonobalanus*, *Quercus*, *Castanopsis*, *Castanea*, and *Lithocarpus*, is more or less similar to the earlier phylogenetic outcome, although the constituent species in Quercoideae were different [34]. Our study confirmed the effectiveness of the chloroplast genome in establishing the monophyletic origin of *L. dealbatus*, indicating the absence of the chloroplast capture phenomenon and interspecific hybridisation, as reported in *Quercus* species by Yang et al. [19]. Further, our phylogenetic analysis using the chloroplast genome confirmed the non-monophyletic origin of *Quercus* species, which is in conformity with Zhou et al. [6]. Since nuclear genome phylogenetic analysis resulted in the monophyletic origin of *Quercus* [6], such contrasting observations necessitate the phylogenetic lineage analysis for *Lithocarpus*, using the nuclear genome for generalising the phylogenetic origin in Quercoideae. 

## 5. Conclusions

Using MGI technology, the current study effectively assembled and annotated the whole chloroplast genomes of *L. dealbatus*. The gene content and synteny of the *Lithocarpus* chloroplast genome were nearly identical. *L. dealbatus*, *L. balansae*, and *L. hancei* have considerable sequence homology. The analysis of repeat elements indicated small changes in the overall amount of repeat elements among the genomes studied. The ML tree clearly demonstrated that *Lithocarpus* species formed a monophyletic group, and the whole clade of *Lithocarpus* was closely connected to one clade of *Quercus* species. The comparison of *L. dealbatus* chloroplast genomes with those of other Quercoideae species improved our understanding of evolutionary lineage. Furthermore, the mutational hotspot areas identified in the current study may be useful in distinguishing between closely related Quercoideae species. We propose that the highly mutational diverging region be exploited as a possible barcode for better species resolution in diverse Quercoideae taxa. The newly proposed *in silico* barcodes need to be validated in terms of their resolution power for species delimitation and phylogenetic reconstruction. Thus, the presence of these mutation hotspots, i.e., positions with concentrated mutations, have high future usage potential for species or any other taxon delimitation. 

## Figures and Tables

**Table 1:** Details list of genes encoded by *L. dealbatus* chloroplast genome.

Gene Category	Gene Name				
**Ribosomal RNA genes**	*rrn16 ^D^*	*rrn23 ^D^*	*rrn4.5 ^D^*	*rrn5 ^D^*	
**Transfer RNA genes**	*trnA-UGC ^D^* *	*trnC-GCA*	*trnD-GUC*	*trnE-UUC*	*trnF-GAA*
	*trnfM-CAU*	*trnG-GCC ^D^* *	*trnH-GUG*	*trnI-CAU ^D^*	*trnI-GAU ^D^* *
	*trnK-UUU* *	*trnL-CAA ^D^*	*trnL-UAA* *	*trnL-UAG*	*trnM-CAU*
	*trnN-GUU ^D^*	*trnP-GGG*	*trnP-UGG*	*trnQ-UUG*	*trnR-ACG ^D^*
	*trnR-UCU*	*trnS-GCU*	*trnS-GGA*	*trnS-UGA*	*trnT-GGU ^D^*
	*trnT-UGU*	*trnV-GAC ^D^*	*trnV-UAC* *	*trnW-CCA*	*trnY-GUA*
**Large subunit of ribosomal proteins (LSU)**	*rpl2 ^D^* *	*rpl14*	*rpl16*	*rpl20*	*rpl22*
	*rpl23 ^D^*	*rpl32 *	*rpl33*	*rpl36*	
**Small subunit of ribosomal proteins (SSU)**	*rps2*	*rps3*	*rps4*	*rps7 ^D^*	*rps8*
	*rps11*	*rps12 ^D^* *	*rps14*	*rps15*	*rps16* *
	*rps18*	*rps19*			
**DNA dependant RNA polymerase**	*rpoA*	*rpoB*	*rpoC1* *	*rpoC2*	
**Photosystem I**	*psaA*	*psaB*	*psaC*	*psaI*	*psaJ*
**Photosystem II**	*psbA*	*psbB*	*psbC*	*psbD^D^*	*psbE*
	*psbF*	*psbH*	*psbI*	*psbJ*	*psbK*
	*psbL*	*psbM*	*psbN*	*psbT*	*psbZ*
**Cytochrome b/f**	*petA*	*petB* *	*petD*	*petG*	*petL*
	*petN*				
**ATP synthase**	*atpA*	*atpB*	*atpE*	*atpF* *	*atpH*
	*atpI*				
**Protease**	*clpP* **				
**Rubisco**	*rbcL*				
**NADH dehydrogenase**	*ndhA* *	*ndhB ^D^* *	*ndhC*	*ndhD*	*ndhE*
	*ndhF*	*ndhG*	*ndhH*	*ndhI*	*ndhJ*
	*ndhK*				
**Maturase**	*matK*				
**Envelop membrane protein**	*cemA*				
**Subunit of acetyl-CoA-carboxylase**	*accD*				
**C-type cytochrome synthesis gene**	*ccsA*				
**Conserved hypothetical chloroplast open reading frames**	*ycf1 ^D^*	*ycf2 ^D^*	*ycf3* **	*ycf4*	

* Genes with one intron. ** Gene containing two introns. *^D^* Gene harbouring duplicated copies.

**Table 2:** Comparative analysis of seven Quercoideae chloroplast genomes.

Species	*L. dealbatus*	*L. balansae*	*L. hancei*	*C. henryi*	*C. sclerophylla*	*Q. pannosa*	*T. doichangensis*
**Genome size**	161,476 bp	161,020 bp	161,304 bp	160,907 bp	160,497 bp	161,222 bp	159,938 bp
**LSC**	90,732 bp	90,596 bp	90,585 bp	90,527 bp	90, 255 bp	90,522 bp	89,445 bp
**SSC**	18,987 bp	19,160 bp	18,959 bp	18,998 bp	25,675 bp	19,000 bp	19,295 bp
**IR**	25,879 bp	25,632 bp	25,880 bp	25,961 bp	18,892 bp	25,850 bp	25,600 bp
**Protein coding genes**	86	87	87	82	86	85	81
**rRNAs**	8	8	8	8	8	8	8
**tRNAs**	39	39	44	37	37	37	39
**Duplicated genes**	21	19	24	17	21	17	17
**CDS**	80,577 bp	80,142 bp	80,199 bp	77,685 bp	79,647 bp	78,852 bp	71,778 bp
**NCDS**	80,899 bp	80,878 bp	81,105 bp	83,222 bp	80,850 bp	82,370 bp	88,360 bp
**GC%**	36.7	36.7	36.7	36.7	36.8	36.9	37

CDS—coding sequence. NCDS—Noncoding sequence.

## Data Availability

The assembled genome can be accessed in NCBI database with accession number MZ322408.

## Supplementary Materials

The following supporting information can be downloaded at: https://www.mdpi.com/article/10.3390/life12060828/s1. Table S1. Comparison of RNA editing sites and genes in *L. dealbatus* and Quercoideae chloroplast genomes. Figure S1. Enlarge view of inversion present in the *T. doichangensis* chloroplast genome shown in green colour box. Figure S2. Comparison of the DNA diversity (*Pi*) values of non-coding regions for A. *Lithocarpus*, B. *Quercus*, C. *Castanea* and D. *Castanopsis*. Figure S3. Divergence time of different members of Quercoideae clade.
