# Supplementary figures and images for: Chloroplast Genome of Lithocarpus dealbatus (Hook.f. & Thomson ex Miq.) Rehder Establishes Monophyletic Origin of the Species and Reveals Mutational Hotspots with Taxon Delimitation Potential
Source: Life (Basel). 2022 Jun 2;12(6):828. doi: 10.3390/life12060828 (PMC9225305; doi:10.3390/life12060828)

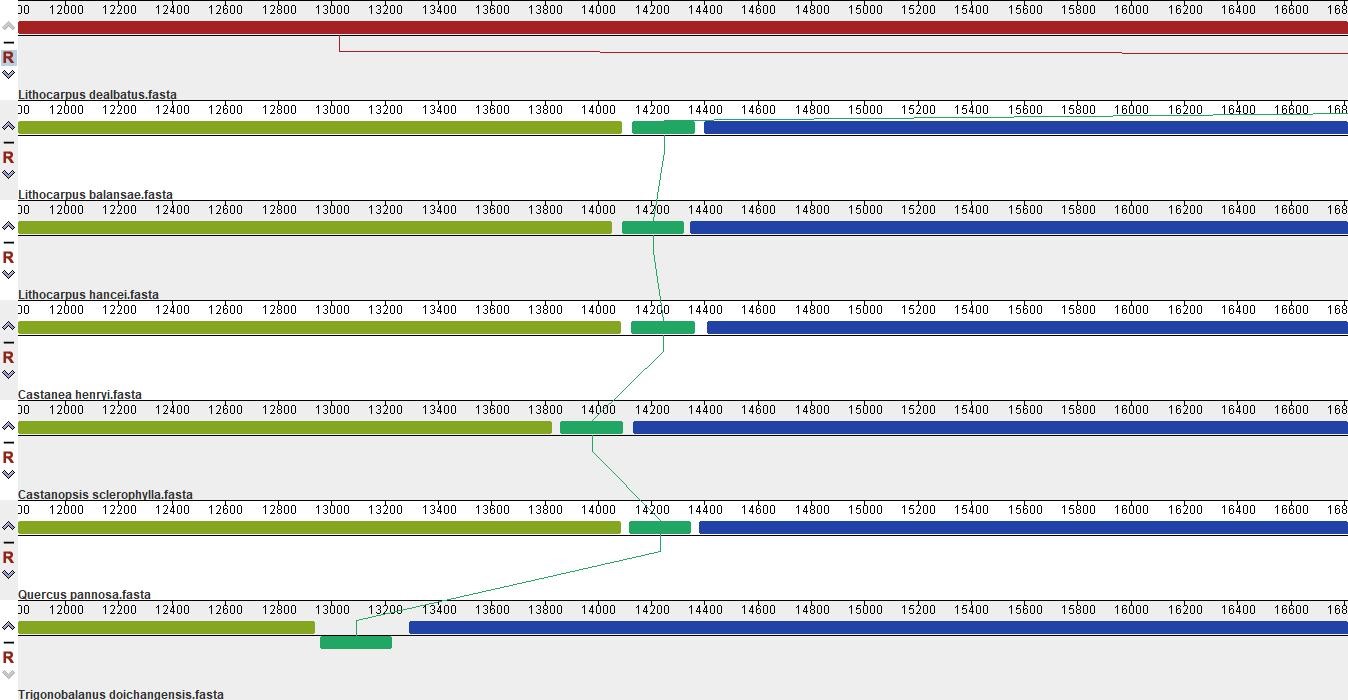

Supplement: Supplementary file 1 [file life-12-00828-s001.zip › Figure S1.jpg]

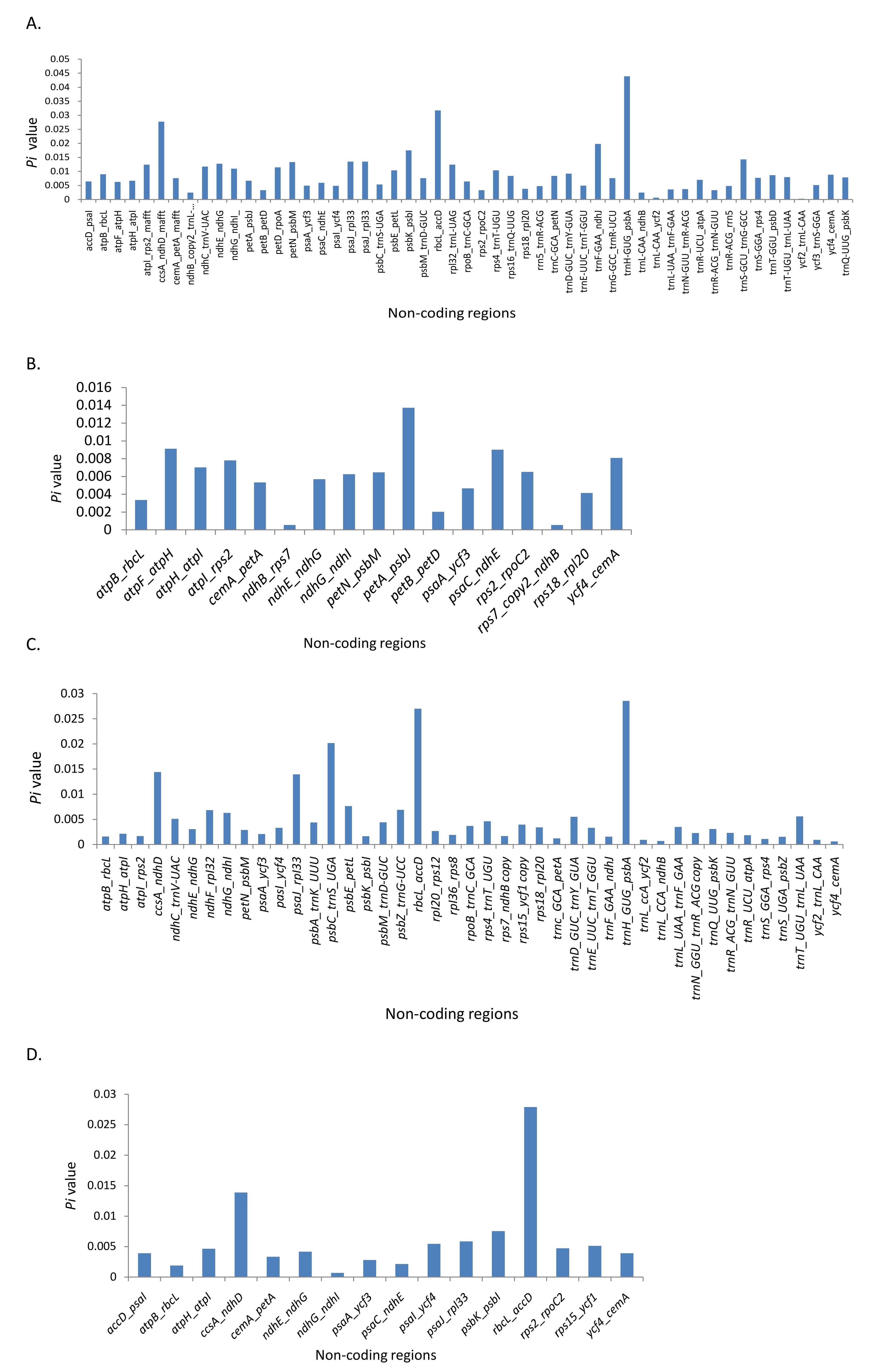

Supplement: Supplementary file 1 [file life-12-00828-s001.zip › Figure S2.jpg]

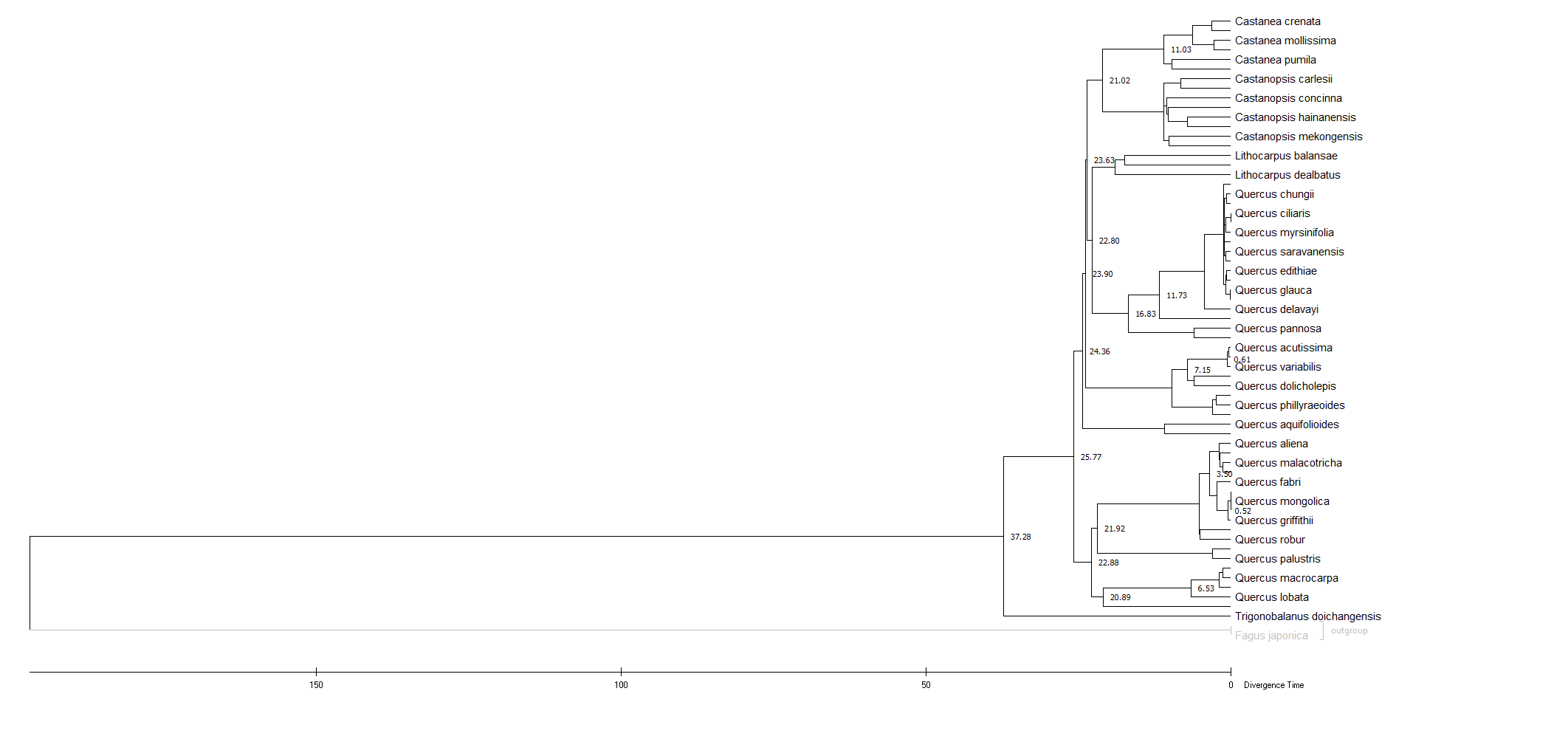

Supplement: Supplementary file 1 [file life-12-00828-s001.zip › Figure S3.png]
